# Identification of a Twelve-microRNA Signature with Prognostic Value in Stage II Microsatellite Stable Colon Cancer

**DOI:** 10.3390/cancers15133301

**Published:** 2023-06-23

**Authors:** Ferran Moratalla-Navarro, Anna Díez-Villanueva, Ainhoa Garcia-Serrano, Adrià Closa, David Cordero, Xavier Solé, Elisabet Guinó, Rebeca Sanz-Pamplona, Xavier Sanjuan, Cristina Santos, Sebastiano Biondo, Ramón Salazar, Victor Moreno

**Affiliations:** 1 Oncology Data Analytics Program, Catalan Institute of Oncology (ICO), 08908 Barcelona, Spain; 2 Colorectal Cancer Group, Bellvitge Biomedical Research Institute (IDIBELL), 08908 Barcelona, Spain; 3 Consortium for Biomedical Research in Epidemiology and Public Health (CIBERESP), 28029 Madrid, Spain; 4 Department of Clinical Sciences, Faculty of Medicine, University of Barcelona (UB), 08907 Barcelona, Spain; 5 Department of Clinical Science, Intervention and Technology (CLINTEC), Karolinska Institutet, 14186 Stockholm, Sweden; 6 Department of Pathology, Netherlands Cancer Institute, 1066 CX Amsterdam, The Netherlands; 7 Molecular Biology CORE, Center for Biomedical Diagnostics, Hospital Clinic de Barcelona, 08036 Barcelona, Spain; 8 Translational Genomic and Targeted Therapeutics in Solid Tumors, August Pi i Sunyer Biomedical Research Institute (IDIBAPS), 08036 Barcelona, Spain; 9 Lozano Blesa University Hospital, Aragon Health Research Institute (IISA), Aragon I+D Foundation (ARAID), Government of Aragon, 50009 Zaragoza, Spain; 10 Department of Pathology, Bellvitge University Hospital, 08907 Barcelona, Spain; 11 Oncology Service, Catalan Institute of Oncology (ICO), 08908 Barcelona, Spain; 12 Consortium for Biomedical Research in Oncology (CIBERONC), 28029 Madrid, Spain; 13 Department of General and Digestive Surgery, Bellvitge University Hospital, 08907 Barcelona, Spain

**Keywords:** colorectal cancer, microRNA, prognosis, biomarker

## Abstract

**Simple Summary:**

Colorectal cancer (CRC) is one of the most prevalent cancers, and approximately a quarter of patients diagnosed at stage II exhibit a significant risk of recurrence. In this study, we successfully identified a microRNA (miRNA) signature allowing the recognition of patients at high recurrence risk. The validity of these findings has been confirmed through an entirely separate group of patients diagnosed with stage II microsatellite stability (MSS) colon adenocarcinoma (COAD). Most of the miRNAs present in the signature have demonstrated prognostic relevance in various other cancer types. Upon examining their gene targets, we discovered that some of these miRNAs are intricately involved in pivotal pathways of cancer progression.

**Abstract:**

We aimed to identify and validate a set of miRNAs that could serve as a prognostic signature useful to determine the recurrence risk for patients with COAD. Small RNAs from tumors of 100 stage II, untreated, MSS colon cancer patients were sequenced for the discovery step. For this purpose, we built an miRNA score using an elastic net Cox regression model based on the disease-free survival status. Patients were grouped into high or low recurrence risk categories based on the median value of the score. We then validated these results in an independent sample of stage II microsatellite stable tumor tissues, with a hazard ratio of 3.24, (CI_95%_ = 1.05–10.0) and a 10-year area under the receiver operating characteristic curve of 0.67. Functional analysis of the miRNAs present in the signature identified key pathways in cancer progression. In conclusion, the proposed signature of 12 miRNAs can contribute to improving the prediction of disease relapse in patients with stage II MSS colorectal cancer, and might be useful in deciding which patients may benefit from adjuvant chemotherapy.

## 1. Introduction

CRC is the third most newly diagnosed cancer type worldwide. Although systematic screening programs have reduced the incidence of CRC in Western countries [1], it is still the second leading cause of cancer-related deaths worldwide for both men and women [2], with millions of cases being reported each year. Currently, stage at diagnosis is the most relevant predictor of prognosis. It is known that about a quarter of patients with CRC are diagnosed in stage II, with localized disease and no evidence of regional lymph node invasion [3]. Nevertheless, disease will recur or progress to distant metastasis in about 20–25% of these patients. Clinical and pathological risk factors, such as the size and location of the tumor, are used to identify patients at high risk of recurrence, but they are not always reliable. As a result, there has been growing interest in the use of biomarkers to improve the accuracy of prognostic and predictive testing for CRC patients [4,5,6,7,8]. However, these approaches are limited by small populations or accuracy [9,10]. Molecular biomarkers could be used to identify patients who are at high risk of disease recurrence or improve stratification of patients who could benefit from adjuvant chemotherapy or immunotherapy.

miRNAs are short, double-stranded, non-coding RNA molecules, typically between 19 and 24 nucleotides in length, which play a critical role in the regulation of gene expression. MiRNAs are involved in post-transcriptional regulation of multiple protein coding genes, mainly by binding to the 3’ untranslated regions (UTRs) of target genes, leading to inhibition of messenger RNA (mRNA) transcription [11]. Changes in miRNA expression affect target genes regulation, and consequently, their deregulation can lead to irregular cell processes related to tumor development and progression [12]. To date, several miRNAs have been proposed to be either oncogenic or tumor suppressors [13,14,15], and there have been some miRNA signatures proposed as molecular biomarkers in CRC for both diagnosis and prognosis, as well as treatment decisions [16,17,18,19].

In this study, we aimed to identify and validate a signature of miRNAs with prognostic value in stage II COAD patients. We used next-generation sequencing (NGS) techniques to obtain miRNA expression values for a set of tumor samples, and we tried to validate the findings in an independent sample series.

## 2. Materials and Methods

### 2.1. Subjects and Samples

In the discovery series, we included Colonomics (CLX): 98 tumor tissue samples, MSS stage II patients with a new diagnosis of COAD at the University Hospital of Bellvitge in Barcelona (Spain) between January 1996 and December 2000. Patients were selected from those that had donated fresh tissue to the biobank and had undergone a complete surgical resection of the tumor, but had not received adjuvant chemotherapy. In addition, a minimum of 3 years of follow-up was required.

The validation series included public independent samples of 130 COAD patients (stage II) from The Cancer Genome Atlas (TCGA) study.

The study was performed in accordance with relevant ethics guidelines and regulations. The Clinical Research Ethics Committee of the Bellvitge Hospital approved the study protocol (PR178/11). Individuals provided written informed consent to participate and for genetic analysis to be carried out on their samples. Additional information about the study can be found at www.colonomics.org (accessed on 15 May 2023). This study carefully follows the recommendations for reporting proposed by the REMARK guidelines [20].

### 2.2. Sample Processing

Tumor samples were cut by the pathologist from the surgical specimen during the first hour after removal and kept frozen at −80 °C in the hospital’s tumor bank. Total RNA was isolated from tissue samples using the miRCURY^TM^ RNA isolation kit (Exiqon, Vedbaek, Denmark) according to the manufacturer’s protocol, quantified using a NanoDrop® ND-1000 Spectrophotometer (Nanodrop technologies, Wilmington, DE, USA) and stored at −80 °C. The quality of these RNA samples was assessed with the RNA 6000 Nano Assay (Agilent Technologies, Santa Clara, CA, USA) following the manufacturer’s recommendations. The RNA integrity number (RIN) showed high quality values for all the samples (mean = 7.89, sd = 0.86). The RNA purity was measured with the ratio of absorbance at 260 nm and 280 nm (mean = 1.96, sd = 0.04). The quality control for the small RNA fraction was assessed with the Small RNA Assay in the Agilent 2100 Bioanalyzer (Agilent Technologies, Santa Clara, CA, USA) following the manufacturer’s recommendations.

### 2.3. Small RNA-seq Analysis of the Discovery Series

The small RNA-seq was performed through the SOLiD platform. The PureLink miRNA isolation kit was used to construct the libraries of compatible fragments with SOLiD from an enriched fraction of small RNA. Sequencing microspheres were obtained by applying an emulsion PCR into an equimolar mixture of 48 libraries followed by an enrichment process before charging in the reaction chamber. Finally, the reaction to obtain the sequences (35 nucleotides + 10 nucleotides barcode) from the small RNA fraction was performed with the Applied Biosystems SOLiD 4 System. Samples were randomly distributed among the different sequencing slides to minimize batch effects. The data quality was estimated using the SOLiD Experimental Tracking System (SETS) software.

### 2.4. Expression Data of the Discovery Series

CLX is a multiomics experiment design with different high-throughput sequencing data. In addition to small RNA-seq, it has microarray expression data for the same subjects. Sample processing, quality control and normalization are described elsewhere [21].

### 2.5. Bioinformatics Analysis

Quality control of sequenced reads was ensured using specially designed bioinformatics framework for the SOLiD system [22]. Total number of reads, proportion of miscalled reads and low-average-quality-score proportion reads were evaluated. All samples passed the quality control criteria and were selected for further analysis. Next, quantification of specific miRNAs was performed by mapping reads to the reference of mature miRNA sequences annotated in miRBase release 22 [23], containing 2641 human mature miRNA sequences. The FASTX-toolkit [24] was used to preprocess miRNA data and provide compatible sequences for mapping with Bowtie aligner. Read adapters were trimmed with cutadapt [25], and finally, a table of counts was generated with SAMtools [26]. A principal component analysis (PCA) was computed to detect possible outliers. A filter based on low variability of miRNAs across all samples was performed to remove unwanted noisy data (standard deviation < 0.1). Data normalization was performed using DESeq2 package [27], after which it was transformed with a logarithmic function to reduce positive skewness. MiRNAs with normalized expression values not detected in more than 90% of samples were filtered out due to low expression. This criterion was mandatory for both discovery and validation datasets.

### 2.6. Statistical Analysis of Prognosis

For this study, disease-free survival (DFS) was assessed, and disease progression, defined as local tumor recurrence, metastasis or cancer-related death, was the event of interest. First, we wanted to inspect miRNA profiles based on possible sources of confounding variables. For this purpose, differential miRNA expression analysis (DEA) was carried out with the DESeq2 package for sex and tumor site. In addition, a proportional hazards assumption test was performed to assess possible sources of analysis bias from common covariates: age, sex and tumor site (left or right colon). Univariate Cox proportional hazard models were computed for each miRNA and adjusted for age, sex, tumor site and sub-stage. Kaplan–Meier survival curves were used to graph the results, which were split by median normalized expression values. Next, in order to identify an miRNA signature that could capture disease progression, a regularized Cox regression model was performed with an alpha parameter (α = 0.5) and leave-one-out cross-validation. Adjustment variables were included in all models, and we selected the model that minimized the cross-validation error. The coefficients for the miRNAs that were not shrunk in the optimal model were used to compute an miRNA prognosis risk score (RS) as follows:

$$\sum_{i=1}^{n}=\beta_{i}*\;expr_{i}$$

where *n* is the total number of active miRNAs in the model, *β_i_* is the coefficient for each active miRNA, and *expr_i_* is the expression value of each active miRNA. The score obtained was ranked and split into two equal groups by the median value. The performance of the model was assessed with Cox proportional hazard models (selecting miRNA (RS) and adjustment variables, as mentioned before) and Kaplan–Meier survival curves. Univariate Cox proportional hazard models and Kaplan–Meier estimates were obtained with the *survival* R package [28]. Regularized Cox regression models were computed with the *glmnet* R package [29]. All statistical analysis was performed in R version 3.5 [30].

### 2.7. Validation Analysis

For the validation dataset, TCGA COAD samples were filtered in order to obtain similar clinical characteristics and reproducible analysis. Sample exclusion criteria included no clinical information for disease-free survival status or microsatellite instability (MSI) subtype, the latter of which was assessed in cBioPortal [31,32]. Normalization of miRNA expression values and filtering was identical in discovery and validation series. Independent prognosis analysis for stage II was assessed. The same coefficients and cutoffs obtained in the training dataset were used for the validation.

### 2.8. Functional Characterization

We conducted two separate approaches to characterize the resulting miRNA signature and score. On the one hand, for the signature study, two different miRNA–mRNA interaction resources were interrogated to extract high-confidence gene targets for each miRNA present in our signature. Only common interactions present in mirDB (release 6.0) [33,34] and miRTarBase (release 9.0) [35] were included to define the functional role of miRNAs associated with prognosis. mirDB annotates predicted miRNA–target interactions (MTI) while miRTarBase captures experimentally validated MTIs from research articles. Network analysis was carried out with igraph [36] to identify hub miRNAs; next, we performed an enrichment analysis with the ReactomePA [37] R package based on the REACTOME pathway database, including direct targets for each selected miRNA. On the other hand, we wanted to study the relationship between miRNA prognosis RS and the abundance of tissue-infiltrating immune cell populations that could potentially play different roles in the tumor microenvironment. For this purpose, a deconvolution method [38] was used to estimate cell population proportions (immune and non-immune stromal) from average gene expression signals. Non-parametric Spearman’s rank-order correlations were computed to evaluate correlation patterns between each of the ten different cell types and miRNA prognosis RS.

## 3. Results

### 3.1. Study Population Characteristics and Quality Control of Samples

The CLX small-RNA sequencing dataset comprised 100 tumor tissues, stage II MSS, and 2641 different miRNAs were initially identified. The first filter removed 153 miRNAs due to low variability. The second filter was applied to remove low-expression features. A total of 928 miRNAs passed the filtering criteria. Two samples were filtered out after PCA analysis (see Supplementary Figure S1). The same procedure was performed for the validation dataset, which lowered the number of different miRNAs from 2117 to 796. Finally, 605 miRNAs were determined to be present in both datasets and were selected for the next analysis. Table 1 summarizes the main characteristics of the patients included in both datasets. DEA comparing tumor location resulted in only 10 significant miRNAs with different profiles between the left and right sides (seven overexpressed on the right side, three overexpressed on the left side). All of them had an absolute log2-fold change greater than 0.5. When comparing expression profiles between sex, only two significant miRNAs were observed (Supplementary Table S1 and Figure S2). None of the inspected covariates violated the proportional hazards assumption of the Cox model (*p*-values > 0.05, Supplementary Table S2). Univariate Cox proportional hazard models, adjusted by sex, age, site and stage, identified 55 miRNAs associated with disease-free survival (*p*-value < 0.05). However, none of them passed the false discovery rate (Benjamini–Hochberg-adjusted *p*-value < 0.05) (Supplementary Table S3).

### 3.2. miRNA Signature and Score

The regularized Cox regression model resulted in a 12-miRNA signature computed in the discovery dataset (Table 2). Of note, all miRNAs present in the signature were statistically significant according to the univariate models (*p* < 0.05), and all of them showed the same trend at the individual coefficient signs, suggesting low collinearity between all of them (Supplementary Table S4), which was confirmed with a Spearman’s correlation matrix (absolute Spearman’s r ≤ 0.43 for all pairwise comparisons (Supplementary Figure S3). 

The RS formula obtained was:

miRNA RS = hsa-miR-1185-5p × (−0.185) + hsa-miR-16-5p × (−0.111) + hsa-miR-181a-2-3p × 0.181 + hsa-miR-204-5p × 0.003 + hsa-miR-2355-3p × 0.242 + hsa-miR-29b-2-5p × (−0.306) + hsa-miR-331-3p × 0.153 + hsa-miR-423-3p × (−0.355) + hsa-miR-432-5p × (−0.187) + hsa-miR-497-5p × (−0.183) + hsa-miR-656-3p × (−0.526) + hsa-miR-935 × (−0.136)

**Figure 1:**
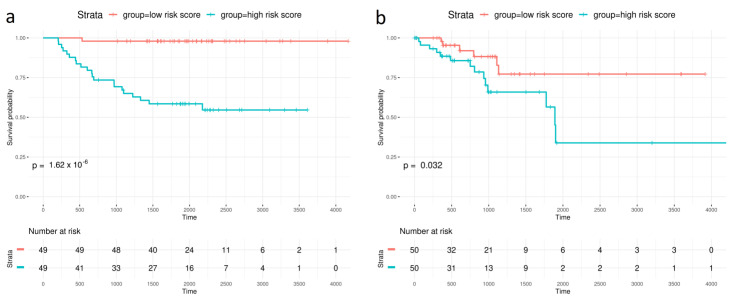

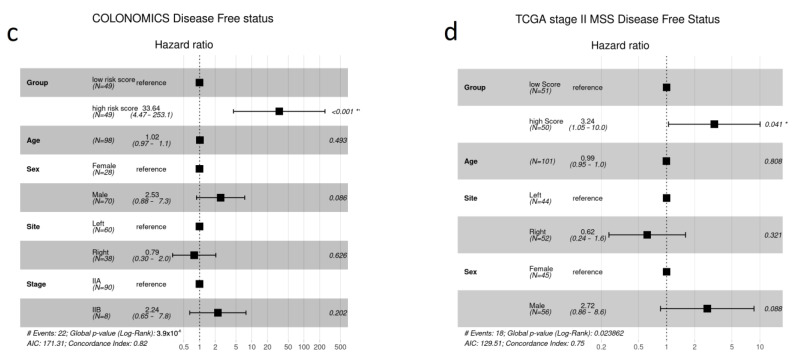
Comparison of the prognostic value with stratification analysis by miRNA risk group using Kaplan–Meier disease-free survival curves. (**a**) Discovery series (Colonomics). (**b**) Validation series (TCGA stage II MSS). Multivariate COX regression hazard models. (**c**) Discovery series (Colonomics). (**d**) Validation series (TCGA stage II MSS). Statistically significant HRs denoted with *.

**Figure 2:**
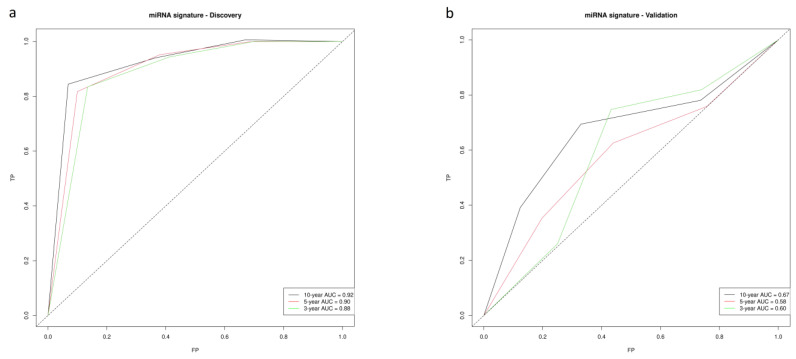
ROC curves at 3-, 5- and 10-year disease-free survival. (**a**) Discovery series (Colonomics). (**b**) Validation series (TCGA stage II MSS).

Kaplan–Meier curves demonstrated a good performance of the model, clearly differentiating low- and high-risk patient log-rank *p*-values = 1.62 × 10^−6^. Patients in the high-risk group were found to have higher recurrence rates (HR = 33.59, 4.34–244.8, *p* < 0.001) (Figure 1a,c). Three-, five- and ten-year disease-free survival (DFS) were selected to compute the area under the ROC curve (AUC). AUCs of 0.89, 0.92 and 0.94 were obtained for three-, five- and ten-year DFS, respectively (Figure 2a).

### 3.3. Validation

CLX miRNA signatures were tested on an independent COAD dataset from TCGA. Overall, the miRNA predictive capacity was poor, which is mainly explained by the poor performance on stage II MSI tumor samples. However, it improved substantially when only stage II MSS samples were analyzed (n = 100). A similar trend to that in CLX was observed; the miRNA risk group had an HR of 3.24, with a range of 1.05–10.0, and *p* = 0.041 (Figure 1d), and the low- versus high-risk patient log-rank *p*-value was 0.034, as assessed using KM curves (Figure 1b). The AUCs were 0.60, 0.59 and 0.67 for three-, five- and ten-year DFS, respectively (Figure 2b).

### 3.4. Functional Characterization

**Figure 3:**
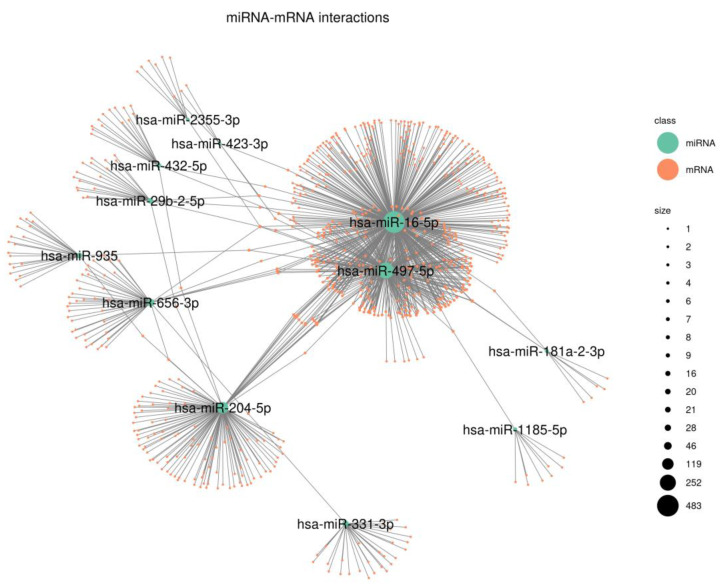
mRNA–miRNA interaction network representation. Light-green nodes represent miRNAs present in the signature, and orange nodes represent their direct target mRNAs. Gray lines represent an interaction present between an miRNA and an mRNA (validated in miRTarBase and also predicted by mirDB). Node size is proportional to its degree centrality measure (number of direct interactors).

Gene target candidates for each miRNA present in our signature were retrieved from mirDB and mirTarBase (10,352 and 3673, respectively). Overall, common MTIs from both datasets revealed 1015 MTIs. Network analysis showed three hub miRNAs: hsa-miR-16-5p (483 gene interactions), hsa-miR-497-5p (252 gene interactions) and hsa-miR-204-5p (119 gene interactions). Of note, the first two miRNAs shared 242 common gene interactions (Figure 3). Next, Reactome pathway analysis identified relevant cancer pathways associated with both hsa-miR-16-5p and hsa-miR -497-5p, such as signaling by the TGF-beta receptor complex (*p* = 1.84 × 10^−7^, *p* = 1.76 × 10^−5^, respectively), regulation of RUNX1 expression and activity (*p* = 2.82 × 10^−7^, 1.47 × 10^−7^, respectively) and aberrant regulation of the mitotic G1/S transition in cancer due to RB1 defects (*p* = 2.82 × 10^−7^, 1.46 × 10^−7^, respectively). The complete results of the enrichment analysis are available in Supplementary Tables S5–S13.

MCP-counter was applied to gene expression data from the discovery series. Abundances of reported immune-infiltrating cell populations, as well as other non-immune cell types, are summarized in Supplementary Figure S4. B-cell infiltrates (Spearman’s r = −0.34, *p*-value = 6.24 × 10^−4^), myeloid dendritic cell infiltrates (Spearman’s r = −0.32 *p*-value = 1.51 × 10^−3^) and T-cell infiltrates (Spearman’s r = −0.29 *p*-value = 3.79 × 10^−3^) appeared with a moderate inverse association with miRNA RS (Supplementary Figure S5). It is worth mentioning that all tested cell population abundances were inversely correlated with miRNA RS, suggesting an overall increase in immune infiltration in tumors with lower values for the computed RS.

## 4. Discussion

A comprehensive analysis has been conducted in order to identify an miRNA signature with potential value for stratifying patients into different disease progression risk groups in stage II MSS colon cancer.

Similar to other prognostic signatures previously published [16,17,18,39], our signature demonstrated a significant association. However, its ability to accurately predict which patients will experience recurrence was limited [40]. Nevertheless, this does not imply that the signature is not valuable since it can be used to classify patients into distinct risk groups.

To the best of our knowledge, only 2 of the 12 miRNAs included in the proposed signature have been previously included in other CRC miRNA signatures [41,42]. However, several of them have been associated with CRC development and/or prognosis; some of them have also been associated with tumoral progression in other tissues. Our findings agree with current knowledge concerning the miRNAs present in our signature. Recently, it has been found that overexpression of hsa-miR-16-5p can inhibit CRC cell proliferation, migration, immune modulation and invasion [43,44]. Another recent publication pointed out the relationship between the down-regulation of hsa-miR-16-5p and hsa-miR-497-5p and the progression of endometrial cancer mediated by circular RNA hsa-circ-0011324 [45]. Both miRNAs appeared to have lower expression values in the CLX series high-risk group, as reported in univariate Cox proportional hazard models. hsa-miR-656-3p was also included in one miRNA classifier for tumor recurrence in stage II CRC [42]. Interestingly, it has also been identified as an inhibitor of CRC cell migration in vitro [46]. In contrast, hsa-miR-204-5p has been identified to be negatively associated with CRC progression and chemoresistance [47,48]. This specific miRNA goes in the opposite direction both in the discovery and validation series. Another miRNA present in the signature, hsa-miR-935, has been studied in different cancer types and seems to have different behaviors depending on the targeted tissue, inhibiting or promoting tumor development in glioblastoma, liver and gastric cancer [49,50,51]. Hsa-miR-423-3p was seen to be down-regulated in hepatocellular carcinoma compared to healthy and liver cirrhosis samples [52]. In a recent study conducted on bladder urothelial carcinoma patients, hsa-miR-432-5p was found to be a good biomarker for diagnosis, being under-expressed in tumoral samples [53]. Regarding the remnant miRNAs, one study suggested opposite effects from what we have reported for hsa-miR-181a-2-3p in glioblastoma [54], and two studies found hsa-miR-331-3p to be under-expressed in prostate cancers [55] and CRC [56] compared to healthy groups. Higher levels of hsa-29b-2-5p expression were associated with the staging of esophageal and gastric cancer [57] in TCGA. However, this association was not observed in TCGA COAD stage II samples. No previous studies were found for hsa-miR-2355-3p and hsa-miR-1185-5p related to cancer.

To assess the complex functional interrelationships between the miRNAs and their putative target mRNAs, we analyzed a network of validated miRNA–target gene associations. Our results show a significant enrichment of genes involved in cellular processes relevant for cancer progression, such as cell cycle regulation, interleukin signaling and cell migration [58,59]. Since the information on validated target genes for miRNA is still incomplete, these results might be biased because more well-known miRNAs share more interactions with important cancer genes. However, this behavior is expected to be minimized with the release of the latest updated versions. In addition, to further evaluate the possible effects of the miRNA signature, the immune profile analysis of the miRNA risk groups revealed an overall increased abundance of all types of immune cell populations as measured through deconvolution analyses, suggesting a protective effect of immune infiltration in tumors [60]. Although the role of immune cell infiltrates in cancer progression is complex and context-dependent [61], it is thought that in early CRC stages, immune cells could help to control the growth and spread of cancer cells. However, as the tumor progresses, these immune cells can adapt a pro-tumorigenic role, promoting tumor growth and metastasis [62,63].

Besides the considerable efforts to generate biomarkers for risk assessment based on miRNA expression levels [16,17,18,42], other emerging tools are being investigated as potential prognostic factors for stage II CRC. One such approach involves examining molecular characteristics, including mutations or expression profile alterations in BRAF, KRAS or PIK3CA. These molecular features can aid in determining more targeted treatment strategies for patients [64,65,66]. Another emerging tool, circulating tumor DNA (ctDNA) analysis, has shown promise in identifying minimal residual disease [67], which results in a higher risk of recurrence for patients and may require closer surveillance or additional treatment options. Furthermore, the study of the gut microbiome is also a recent topic of interest for the assessment of both tumor onset and recurrent disease [68,69]. Lastly, exploring the tumor microenvironment [40,70], such as TILs, tumor-associated fribroblasts and stromal characteristics, and understanding the interactions between tumor cells and their microenvironment might provide valuable prognostic information.

This study has several limitations. The most important is the low number of events in both the discovery and the validation series, which is probably related to the good prognosis for early COAD diagnosis. In addition, our results may have been underestimated due to the lack of information regarding the administration of adjuvant chemotherapy or radiation therapy in the validation series. The low number of events together with the limited sample size reduces the statistical power for this kind of analysis; as a result, we saw a trend in the validation cohort with borderline statistical significance. Another limitation of the study is the lack of microsatellite instability (MSI) patients. The proportion of MSI in our hospital was low, around 8%; thus, the CLX series was only composed of MSS patients.

Overall, based on the reported results, this signature could be valuable to stratify MSS stage II COAD patients and identify those that require adjuvant chemotherapy. Additionally, the individual miRNA prognostic data provided in the discovery series contribute to increasing the knowledge on these markers in CRC.

## 5. Conclusions

In summary, we have identified a panel of 12 miRNAs that can be used to stratify prognosis in MSS stage II COAD. These miRNAs have been described to regulate a large list of genes involved in relevant cancer pathways, which reinforces the validity of the panel. Further studies with larger samples sizes are needed to improve our ability to classify patients with recurrence risk in a more general way. 

## Figures and Tables

**Table 1:** Summary of characteristics of the patients included in the study.

	Colonomics n (%)	TCGA n (%)
Number of Patients	98	130
Gender		
Male	70 (71.43%)	69 (53.08%)
Female	28 (28.57%)	61 (46.92%)
Median Age (Years)	71	69
Tumor Site		
Right	38 (38.78%)	75 (57.69%)
Left	60 (61.22%)	50 (38.46%)
Stage		
II-A	90 (91.84%)	99 (76.15%)
II-B	8 (8.16%)	6 (4.62%)
Disease-Free Survival		
No Event	76 (77.55%)	104 (80.00%)
Event	22 (22.45%)	26 (20.00%)
Microsatellite Instability		
MSS	98 (100%)	101 (77.69%)
MSI	0 (0%)	20 (15.38%)
Median Metastatic Lymph Nodes	0 (100%)	0 (100%)
Median Isolated Lymph Nodes	18.5	20.0
Lymphatic Invasion		
Yes	7 (0.07%)	26 (20.00%)
No	86 (87.76%)	92 (70.77%)
Perineural Invasion		
Yes	2 (2.04%)	13 (10.00%)
No	83 (84.69%)	38 (29.23%)

**Table 2:** List of miRNAs present in the signature and the coefficient extracted from the elastic net Cox regression model.

miRNA	Coefficient
hsa-miR-1185-5p	−0.185
hsa-miR-16-5p	−0.111
hsa-miR-181a-2-3p	0.181
hsa-miR-204-5p	0.003
hsa-miR-2355-3p	0.242
hsa-miR-29b-2-5p	−0.306
hsa-miR-331-3p	0.153
hsa-miR-423-3p	−0.355
hsa-miR-432-5p	−0.187
hsa-miR-497-5p	−0.183
hsa-miR-656-3p	−0.526
hsa-miR-935	−0.136

## Data Availability

Full normalized miRNA expression data matrix of the training set and its associated clinical data are available at the project site: https://www.colonomics.org/data (accessed on 15 May 2023). Raw small RNA seq data are available upon request at the European Genome-Phenome Archive under the study ID: EGAS00001002453; dataset ID: EGAD00001004827.

## Supplementary Materials

The following supporting information can be downloaded at: www.mdpi.com/xxx/s1.Supplementary Figure S1: PCA representation for the first two components of CLX normalized miRNA. Supplementary Figure S2: Volcano plots for DEA miRNA for tumor side (a) and sex (b). Supplementary Figure S3: Correlation plot of the 12 miRNA presents in the signature. Supplementary Figure S4: Histogram of ten cell populations abundance detected by MPC counter method. Supplementary Figure S5: Scatter plots for significant correlation between miRNA prognostic RS and B-cells, T-cells and myeloid Dendritic cells detected by MCP-counter. Supplementary Table S1: Differential miRNA expression analysis. Supplementary Table S2: Cox Proportional Hazards assumption test. Supplementary Table S3: Univariate Cox regression models for discovery series, adjusted by age, sex, site and stage. Supplementary Table S4: Univariate Cox regression models for validation series adjusted by age, sex and site. Supplementary Table S5: hsa-miR-16-5p target gene Enrichment. Supplementary Table S6: hsa-miR-432-5p target gene Enrichment. Supplementary Table S7: hsa-miR-497-5p target gene Enrichment. Supplementary Table S8: hsa-miR-432-5p target gene Enrichment. Supplementary Table S9: hsa-miR-181-a-2-3p target gene Enrichment. Supplementary Table S10: hsa-miR-204-5p target gene Enrichment. Supplementary Table S11: hsa-miR-2355-3p target gene Enrichment. Supplementary Table S12: hsa-miR-656-3p target gene Enrichment. Table S13: hsa-miR-935 target gene Enrichment.
