# Supplementary material for: Identification of a Twelve-microRNA Signature with Prognostic Value in Stage II Microsatellite Stable Colon Cancer
Source: Cancers (Basel). 2023 Jun 23;15(13):3301. doi: 10.3390/cancers15133301 (PMC10340741; doi:10.3390/cancers15133301)

SUPPLEMENTARY MATERIAL

Supplementary Figure S1. PCA representation for the first two components of CLX normalized miRNA. In orange, the two samples filtered with a clear outlier behavior.

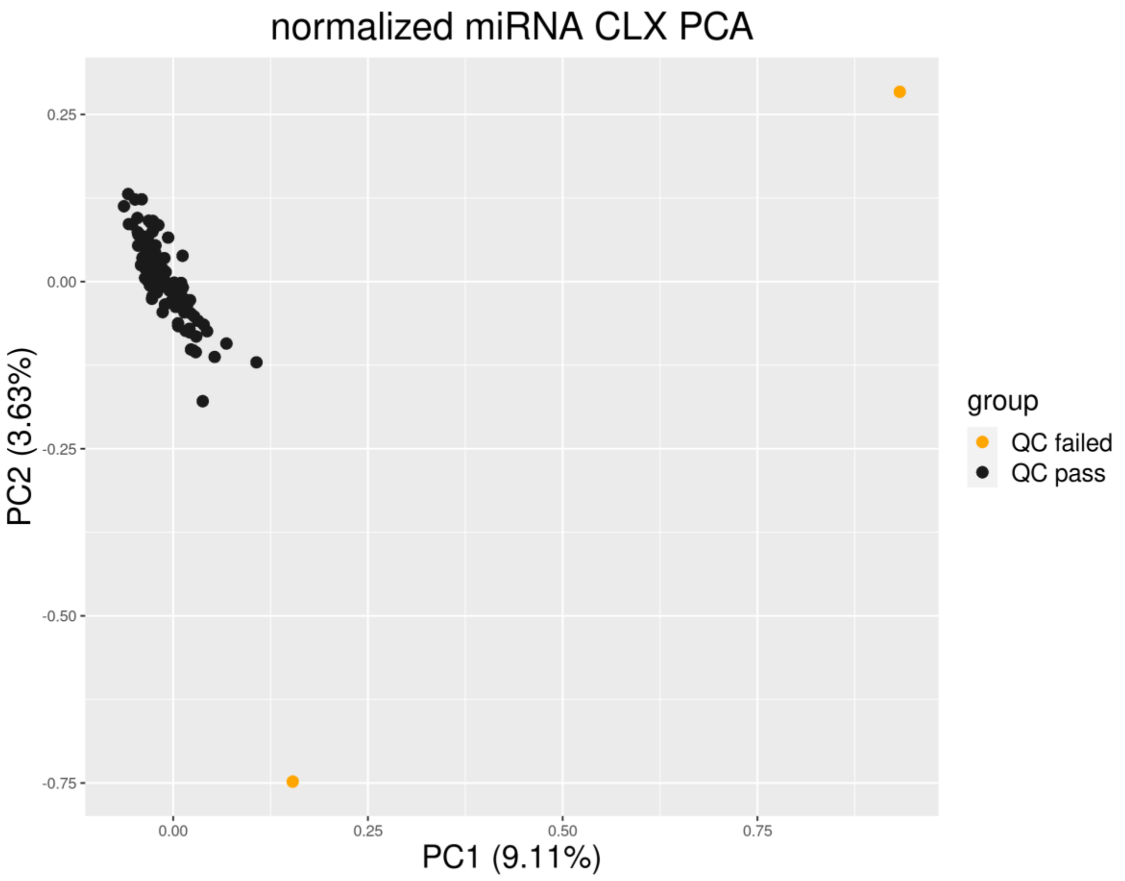

Supplementary Figure S2. Volcano plots for DEA miRNA for tumor side (a) and sex (b). Red dots indicate significant DEAs (adjusted p-value < 0.05)

a)

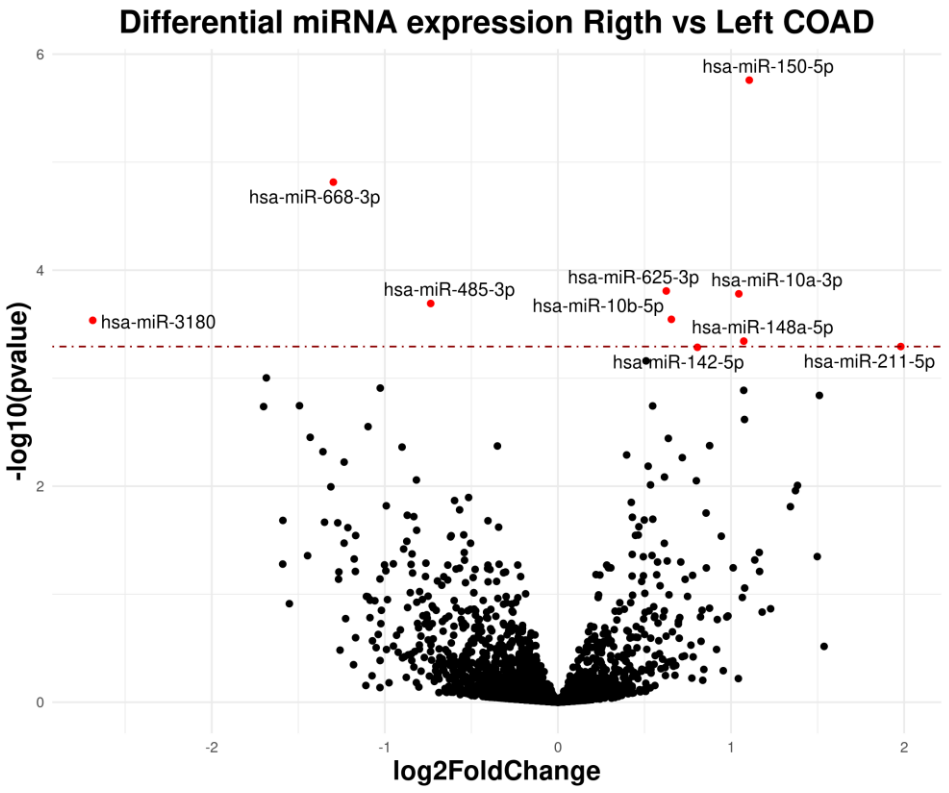

b)

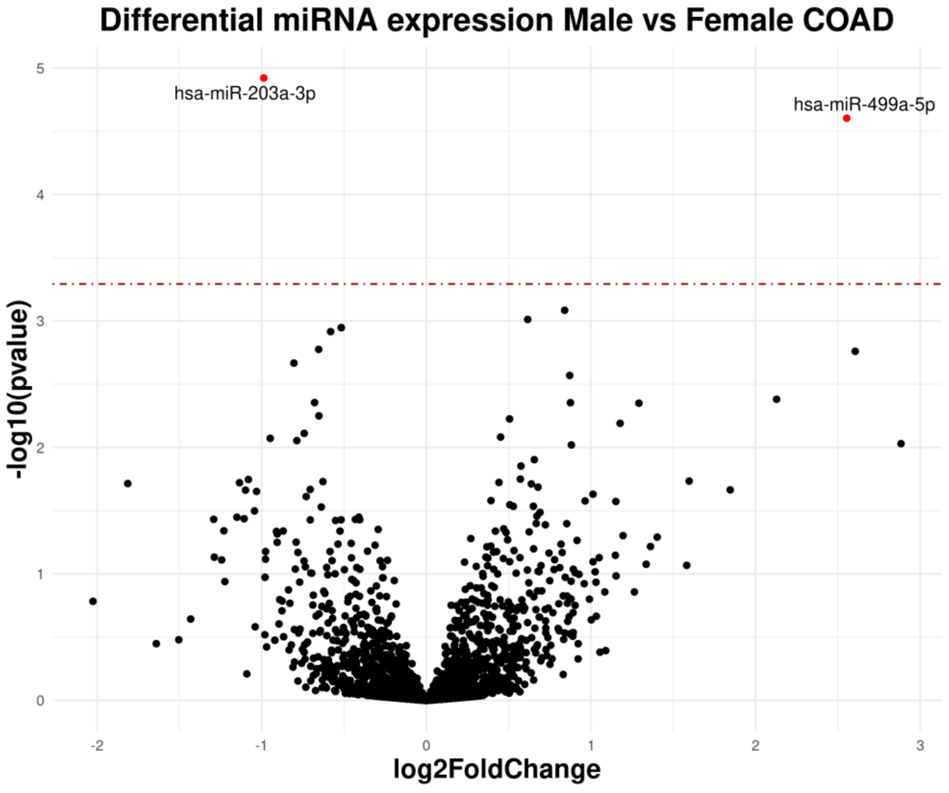

Supplementary Figure S3. Correlation plot of the 12 miRNA presents in the signature. Significant (p<0.05) spearman's Rho values for each pairwise comparison inside the boxes.

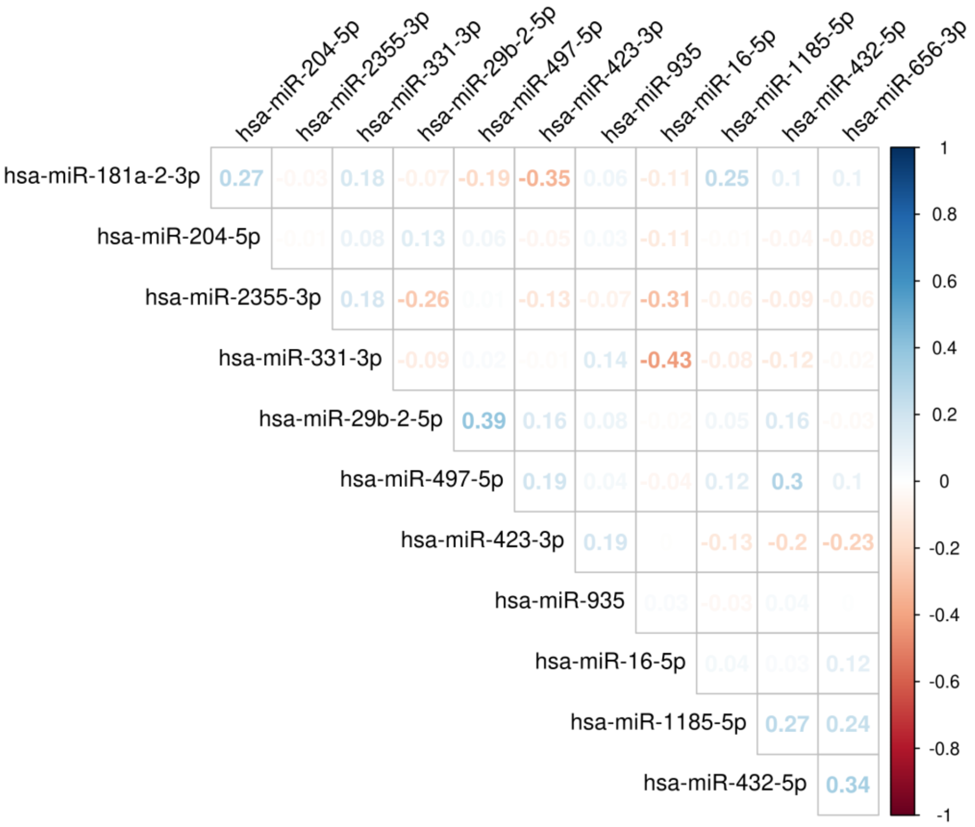

Supplementary Figure S4. Histogram of ten cell populations abundance detected by MPC counter method.

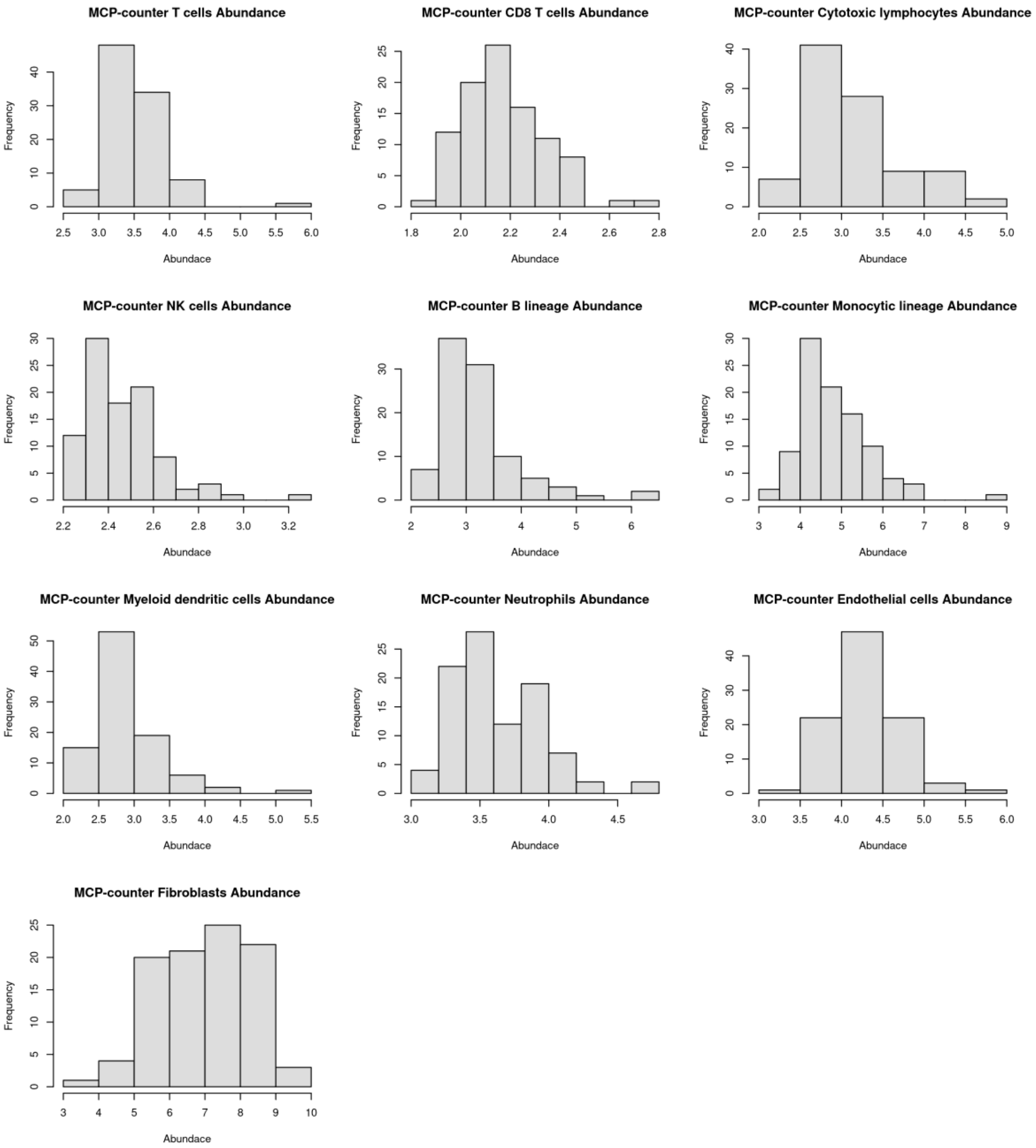

Supplementary Figure S5. Scatter plots for significant correlation between miRNA prognostic RS and B-cells, T-cells and myeloid Dendritic cells detected by MCP-counter

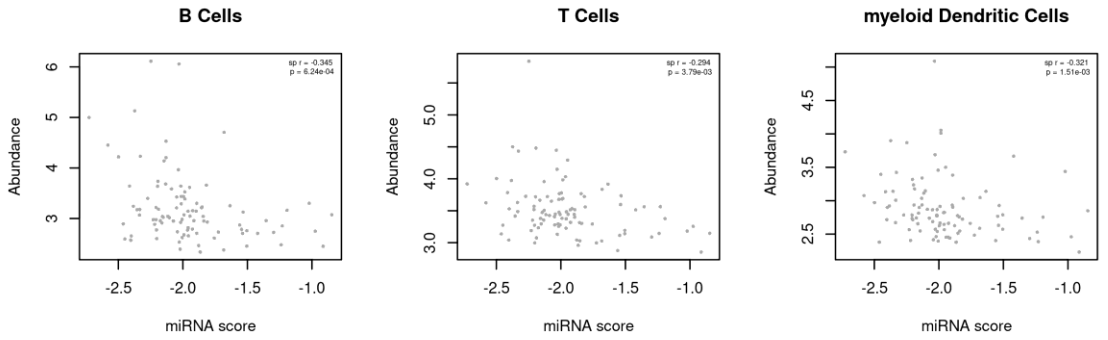

Supplement: Supplementary file 1 [file cancers-15-03301-s001.zip › SupplementaryFigures.pdf]
